# Incidental Finding of the Anomalous Origin of Left Main Coronary Artery from Pulmonary Artery in an Adult Presenting with Arrhythmia-Induced Myocardial Ischemia

**DOI:** 10.1155/2018/6485831

**Published:** 2018-04-01

**Authors:** Shantanu Patil, Mahek Shah, Brijesh Patel, Lohit Garg, Larry Jacobs, Nauman Islam, Matthew Martinez

**Affiliations:** ^1^Department of Medicine, SSM Health St. Mary's Hospital, St. Louis, MO, USA; ^2^Department of Cardiology, Lehigh Valley Hospital, Allentown, PA, USA

## Abstract

Anomalous origin of the left main coronary artery from the pulmonary artery (ALCAPA) is a rare congenital coronary anomaly with high mortality. It is associated with cardiovascular complications and is usually diagnosed soon after birth. Those who survive into adulthood can present with signs of myocardial infarction, heart failure, mitral regurgitation, severe pulmonary hypertension, or sudden cardiac death. We present a 53-year-old female presenting with atrial fibrillation and found to have an incidental diagnosis of ALCAPA who refused surgical correction. We also review the epidemiology, diagnosis, age-based clinical presentations, and treatment options for ALCAPA.

## 1. Introduction

Coronary artery anomalies may be found in up to 1% of the general population [1]. Mostly, these anomalies are benign and can be found incidentally [2]. But certain coronary artery anomalies are associated with life-threatening cardiac complications including heart failure, ischemia, arrhythmias, and death. Anomalous origin of the left main coronary artery from pulmonary artery (ALCAPA), also known as Bland–White–Garland syndrome, is a rare congenital abnormality with high mortality. It causes a left to right shunt, thereby causing “coronary steal” and impaired myocardial perfusion. It is most commonly detected within the first one or two months of life. ALCAPA syndrome is one of the commonest causes of myocardial ischemia and infarction in children. If left untreated, up to 90% of patients with ALCAPA syndrome die within the first year of life [3]. Those who survive into adulthood can present with signs of myocardial infarction, heart failure, mitral regurgitation, severe pulmonary hypertension, or sudden cardiac death. In recent years, reports of adult patients with benign clinical presentation have been described. We present one such adult with undiagnosed ALCAPA syndrome presenting with atrial fibrillation accompanied by significant elevations in cardiac enzymes.

## 2. Case Description

**Figure 1:**
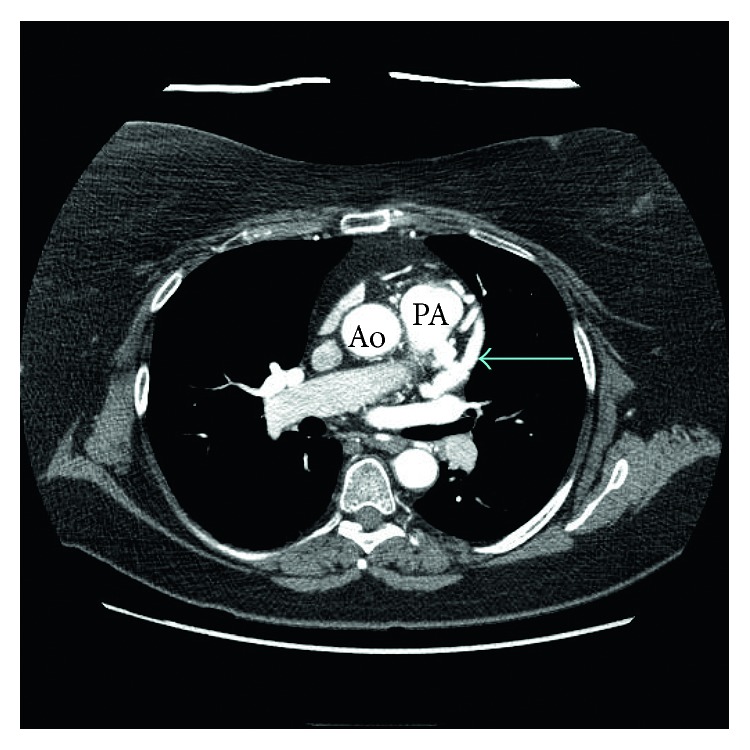
Axial view of multidetector cardiac CT angiogram shows the origin of the left main coronary artery (LMCA) from the main pulmonary artery (blue arrow).

**Figure 2:**
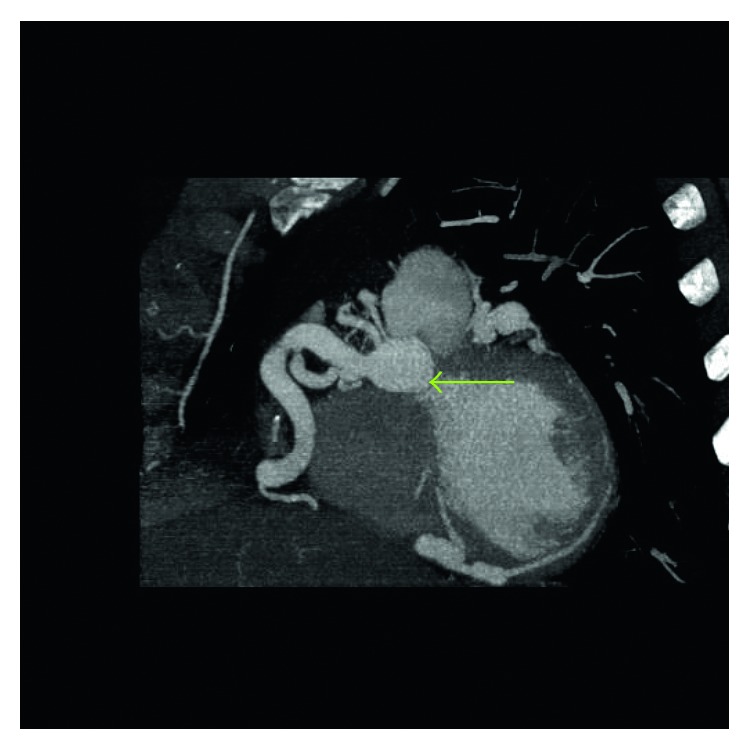
Sagittal view of multidetector cardiac CT angiogram shows an ectatic and dilated right coronary artery (RCA) (green arrow).

A 53-year-old healthy female with known hypothyroidism being treated with thyroid hormone replacement presented to the emergency department with sudden-onset chest pain and palpitations. Her electrocardiogram revealed atrial fibrillation with a ventricular rate of 140 beats per minute. Cardiac auscultation revealed a continuous murmur at the left sternal border. She spontaneously converted to normal sinus rhythm with complete resolution of her chest pain. Her troponin I level was 17 ng/ml. EKG-gated multidetector cardiac computed tomographic angiography revealed the presence of a large anomalous left main coronary arising from the main pulmonary artery (Figure 1). The left anterior descending artery, diagonal branch, and left circumflex arteries arising from the left main were ectatic vessels with extensive collateralization with a large ectatic right coronary artery arising from the right aortic coronary sinus (Figure 2). She remained asymptomatic, and her troponins normalized. Her atrial fibrillation was attributed to iatrogenic hyperthyroidism resulting from an excess in recommended doses for thyroid replacement therapy. The thyroid stimulating hormone level was noted to be low and reported at 0.05 mU/L.

**Figure 3:**
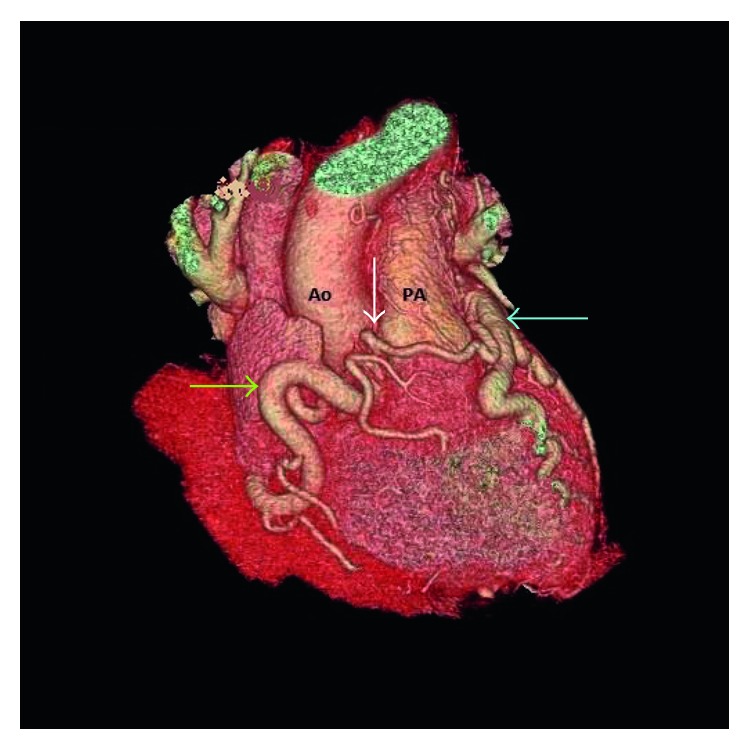
3D reconstruction image of the heart in the right anterior oblique projection demonstrates severely dilated and tortuous RCA (green arrow) and dilated LMCA (blue arrow) along with large intercoronary collaterals (white arrow).

**Figure 4:**
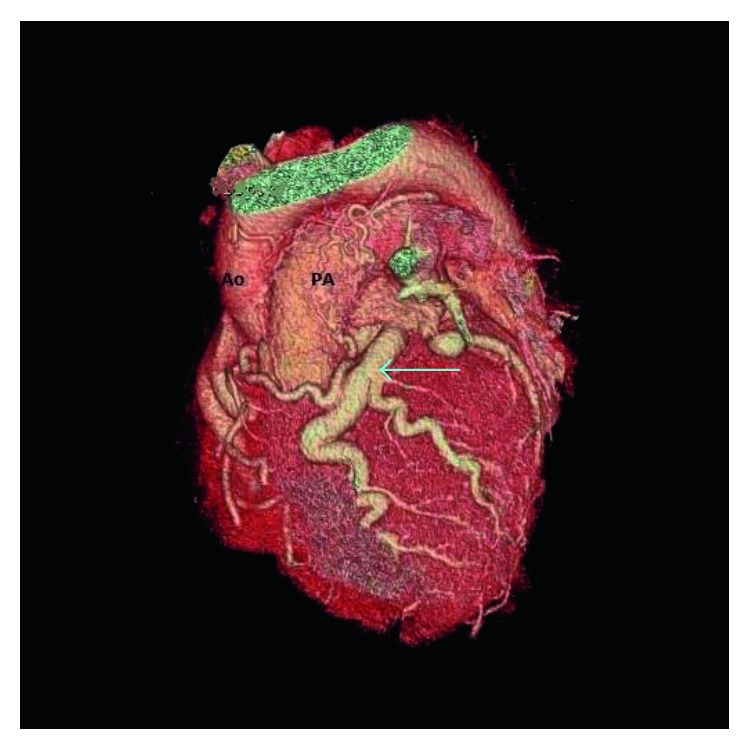
3D reconstruction image of the heart in the left anterior oblique projection demonstrates the dilated LMCA and left anterior descending artery (blue arrow) coursing around the left side of the pulmonary artery.

A 2D echocardiogram revealed normal biventricular size and function with a left ventricular ejection fraction of 55%. Ventricular wall thickness was calculated to be normal in addition to normal left ventricular diastolic function and filling pressures. There were no advanced valvular abnormalities, and a mild degree of mitral and tricuspid regurgitation was present. There was evidence of markedly increased color Doppler signaling within the interventricular septum suggestive of high blood flow through septal collaterals (Figures 3 and 4). Coronary angiography showed evidence of retrograde blood flow in the left coronary system into the pulmonary trunk through collateral supply from the systemic side, secondary to relatively low right-sided pressures, and vascular resistance causing coronary steal (Video 1). Our patient was offered surgery for reimplantation of her left main into the aorta but she refused. Considering that her presentation of myocardial damage was secondary to a lack of oxygenated blood during increased demand at the time of tachycardia, a rhythm control strategy for the atrial fibrillation was adopted and the patient was discharged from the hospital on a reduced dose of thyroid replacement therapy with close follow-up.

## 3. Discussion

Krause first discovered anomalous origin of the left coronary artery from the pulmonary artery in the 1860s, which was later confirmed by Brooks in 1885 during a study on anatomical variations [4, 5]. However, the earliest clinical description was not until 1933 when Bland, White, and Garland observed a 3-month-infant with paroxysmal attacks of acute discomfort precipitated by exertion and associated with profound vasomotor collapse [6]. The EKG findings of this infant mimicked that of myocardial infarction in an adult. An autopsy showed anomalous origin of the left coronary artery arising from the pulmonary artery similar to that previously described by Krause and Brooks. Hence, it is also known as Bland–White–Garland syndrome.

Based on a Canadian heart registry, the incidence of ALCAPA is believed to be 1 per 300,000 live births [7]. ALCAPA occurs due to abnormal division of the conotruncus into the aorta and pulmonary artery or the persistence of the pulmonary buds together with involution of the aortic buds that form the coronary arteries during embryogenesis [8]. During the intrauterine period, the left coronary system has antegrade blood flow from the pulmonary artery, as pulmonary pressure is higher. As pulmonary resistance and oxygen content drops in the pulmonary arterial system during the neonate phase, the myocardium is perfused with blood with low oxygen content. Eventually, collaterals develop from the right coronary artery to supply the branches of the left coronary system, and the pressure in the left coronary system rises. This results in reversal of flow from the left coronary system to low pressure pulmonary system. The phenomenon is often regarded as “coronary steal.” Depending on the extent of collaterals, the clinical spectrum can be divided in four types:

1. *Infantile type*: in a review of postmortem cases, Kaunitz pointed out that closure of ductus arteriosus around 2 months of age was coincidental with development of symptoms [9]. Indeed, closure of ductus arteriosus decreases the oxygenated blood to the left coronary artery via the pulmonary artery. This leads to left ventricular ischemia. About 90% of cases present between 2 and 4 months, with signs of poor growth, tachypnea, profuse sweating, and angina-like episodes (usually occurring after nursing as the sudden onset of a distressed state with pallor). About 90% of those infants who are symptomatic and remain untreated die within first two years of life.
2. *Asymptomatic adult type*: most adults with ALCAPA who survive infancy are found to have extensive collateralization and a dilated RCA. It is hypothesized that in infancy, the RCA supplies not only the posterior but also the lateral wall. These patients have continuous murmur due to collateral blood flow, or mild mitral regurgitation or incidental cardiomegaly on chest X-ray. While initial case reports showed a high percentage of patients present with sudden cardiac death, with recent advent of EKG-gated CT scans, cardiac MRIs, and easy accessibility of echo, higher numbers of asymptomatic and older individuals are being diagnosed in the last few years.
3. *Symptomatic adult type*: these adults present with signs of angina due to increased flow from collaterals to the low-pressure pulmonary artery or coronary steal, which occurs during stress. These patients may also have moderate to severe mitral regurgitation. Mitral regurgitation occurs due to left ventricular dilatation or papillary muscle dysfunction [10].
4. *Sudden cardiac death*: about 85% of adult cases have no previous cardiac symptoms. Histologic studies have shown substantial amount of viable but depressed contractile material and variable degree of fibrosis in myocardial tissue, which is believed to be a precursor to fatal ventricular tachyarrhythmias [11].

A comprehensive review of ECGs by Yau et al. showed Q waves in anterior leads in 50% of patients and left ventricular hypertrophy in up to 28% of all cases [12]. Using Doppler echocardiography, good visualization of the dilated RCA with retrograde flow into the left coronary artery and pulmonary artery along with septal collateralization is diagnostic in 90% of cases. Coronary angiography and cardiac CT scanning can be diagnostic when typical features of echocardiogram are not seen. Cardiac MRI has an advantage of finding scar tissue, areas of ischemia, and myocardial viability [13].

Surgery is the only definitive treatment. While ligation of the LCA at its origin was initially practiced, it was met with high incidence of late sudden cardiac deaths. In modern period, we found an adult case with primary use of the transcatheter closure device to occlude the LCA [14]. Establishing dual coronary supply using saphenous venous graft was found to be superior to ligation of LCA alone [15]. With advent of technology, direct reimplantation of the left coronary artery to the aorta has become very popular with good long-term results. Takeuchi procedure, introduced in 1979, involved the creation of an aortopulmonary window and intrapulmonary tunnel extending from the anomalous ostium to the window. This procedure is now unpopular due to late complications such obstruction of the baffle created between the anomalous coronary artery and supravalvular pulmonary stenosis in up to 33% of cases [16]. Normalization of cardiac function at rest is seen in all operative survivors in the first year itself, albeit exercise testing still may reveal subtle functional disability [17].

Majority of evidence suggests that mild to moderate MR corrects due to reverse LV modeling after the left coronary artery repair. Severe MR caused by irreversible myocardial necrotic damage or papillary muscle infarction needs concomitant repair to prevent long-term MR after alone ALCAPA repair [18]. Implantation of an ICD is usually reserved for patients who present with fatal ventricular arrhythmias. In a small cohort, no recurrence of ventricular arrhythmia was seen up to 10 years after ICD implantation [19].

## 4. Conclusion

ALCAPA is a rare but life-threatening disease. There should a strong clinical suspicion in young patients who present with unexplained cardiomegaly on imaging, continuous murmur on exam, or EKG findings of infarction without a history of ischemia. Due to lack of long-term data on medical therapy, surgical correction is the mainstay of therapy.

## Supplementary Materials

Supplementary Materials Video 1: coronary angiography shows an ectatic right coronary artery supplying the left coronary artery system through collaterals with retrograde flow into the pulmonary artery.
